# High‐Concentration Antibody Formulation via Solvent‐Based Dehydration

**DOI:** 10.1002/adma.202516429

**Published:** 2025-11-23

**Authors:** Talia Zheng, Lucas Attia, Janet Teng, Patrick S. Doyle

**Affiliations:** ^1^ Department of Chemical Engineering Massachusetts Institute of Technology Cambridge MA 02142 USA

**Keywords:** amorphous materials, antibody, hydrogels, precipitation, subcutaneous administration

## Abstract

Although subcutaneous (SC) delivery is the preferred administration route for immunotherapies and other biologics for improved patient compliance and lower healthcare costs, it necessitates high‐concentration antibody formulations. However, high‐concentration antibody solutions face significant instabilities and prohibitively high viscosities. Other approaches for high‐concentration formulations have been developed, including non‐aqueous solutions, which can be irritating or painful, and antibody‐laden hydrogel microparticles, which require centrifugation and are limited to concentrations <300 mg mL^−1^. This work presents a new formulation process wherein the antibody is concentrated and encapsulated into hydrogel microparticles via solvent‐based dehydration. The final dosage form is an aqueous particle suspension with a formulation concentration of 360 mg mL^−1^. In this process, microparticles are synthesized continuously, and antibody precipitation is realized simultaneously to dehydration, which allows for higher antibody concentrations. Antibody phase behavior and precipitation–dehydration kinetics are analyzed. The antibody is structurally and functionally stable in the microparticle post‐processing and after 4 months. Injectability of the suspension meets clinical standards with glide force <20 N. For the first time, an aqueous antibody formulation at high concentrations comparable to non‐aqueous formulations is presented, ideal for subcutaneous administration. The process is envisioned to be generalizable as a platform for SC delivery in multiple clinical applications.

## Graphical abstract

Amorphous solid antibodies are encapsulated into hydrogel microparticles through a solvent‐based dehydration process. The solvent extracts water from antibody solution droplets, causing the antibody to concentrate, and precipitation of the antibody into amorphous solid is induced. The particles are resuspended into aqueous solution in which they are injectable for subcutaneous delivery at high concentrations.

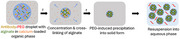

## 1 Introduction

Therapeutic proteins such as monoclonal antibodies (mAbs), which are widely used to treat cancers, auto‐immune diseases, and other chronic and acute illnesses, are typically administered through intravenous (IV) infusion. Recently, subcutaneous (SC) injection has been emerging as the preferred administration route for mAbs due to its reduced cost and healthcare burden, enabling self‐administered injections and improving quality‐of‐life for both patients and caregivers.^[^ ^1^, ^2^ ^]^ However, the injection volume for SC delivery is limited (<2 mL), thus necessitating highly concentrated (>200 mg mL^−1^) formulations to meet the dosing requirements for mAb therapies.^[^ ^3^, ^4^ ^]^ Several strategies have been developed to enable the delivery of high‐concentration biologics, which face molecular instabilities and prohibitively high solution viscosities due to intermolecular interactions, leading to significant delays in clinical trials or product launches because of these formulation difficulties.^[^ ^5^ ^]^ Formulating biologics as solid forms can increase the shelf‐life and stability of the dosage, but can only be reconstituted in aqueous solution at low concentrations.^[^ ^6^, ^7^ ^]^ When reconstituted into non‐aqueous suspensions, both crystalline and amorphous solid mAbs have shown lower viscosities at high loadings compared to in aqueous solution.^[^ ^8^, ^9^ ^]^ However, non‐aqueous formulations, typically solids suspended in an organic solvent, are limited by clinical considerations such as pain and inflammation at the injection site that is especially pronounced for SC injection, as well as regulatory hurdles to be approved for parenteral use.^[^ ^10^, ^11^ ^]^ Thus, an aqueous formulation that maintains the advantages of stability and lower viscosity of the solid suspensions is ideal.

**Figure 1:**
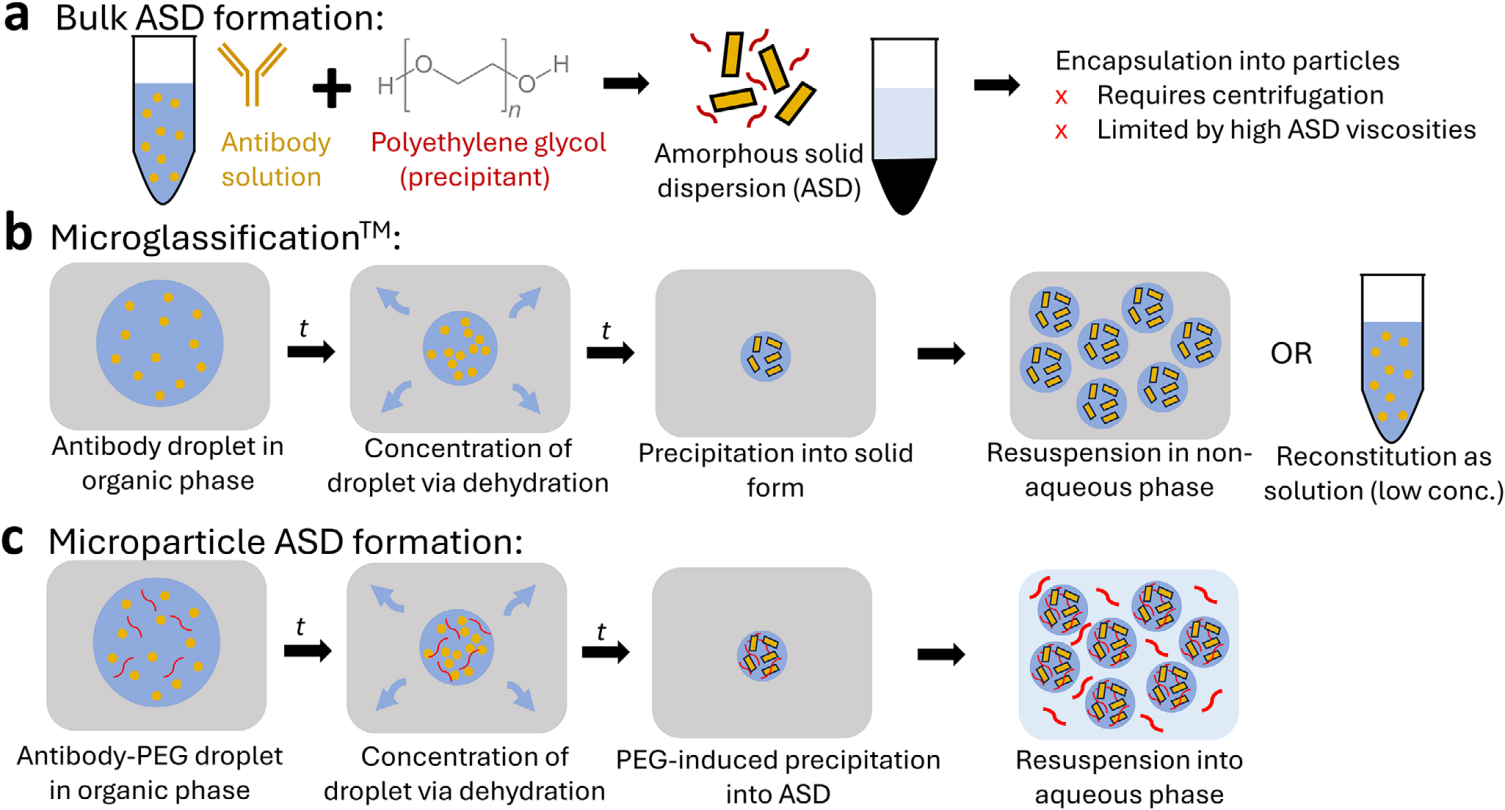
a) Previous process for formulating high‐concentration solid antibodies in aqueous suspension.^[^ ^13^ ^]^ Poly(ethylene) glycol (PEG, a precipitant) is added to antibody solution, inducing precipitation of the antibody into an amorphous solid dispersion (ASD). The ASD is then concentrated and encapsulated into particles via centrifugal dripping, both steps being limited by ASD viscosities at higher concentrations (>300 mg mL^−1^). b) Microglassification, a solvent‐based dehydration process for making solid protein microbeads.^[^ ^14^ ^]^ A droplet of antibody solution is emulsified in an organic phase, extracting water from the droplet and causing precipitation of the antibody into its solid form. These highly concentrated microbeads can then be resuspended in a non‐aqueous phase for delivery or reconstitution into a low‐concentration solution. c) Microparticle ASD formation process proposed in this work. A droplet of antibody solution with precipitant (PEG) is emulsified in an organic phase, extracting water from the droplet and concentrating both antibody and PEG, which induces precipitation of the antibody into an ASD. The ASD is stabilized by PEG and the resulting microparticles can be resuspended at high concentrations in an aqueous phase with PEG for delivery.

Previous work has developed solid (crystalline or amorphous) antibodies, which are loaded at high concentrations into alginate hydrogel microparticles and suspended in aqueous solution with poly(ethylene) glycol (PEG), a well‐established parenteral excipient.^[^ ^12^, ^13^ ^]^ PEG induces controlled precipitation of the protein by steric exclusion effects, and the precipitated solids can then be mixed with alginate and synthesized into hydrogel microparticles. The lubricious hydrogel microparticle reduces viscosity of the formulation and hydrogel matrix masks protein–protein interactions, which are problematic for stability and injectability. Suspending the microparticles in PEG solution ensures that the antibodies are maintained in their solid dispersion form within the hydrogel microparticle. However, the formulation concentrations achieved thus far (≈150–300 mg mL^−1^) have been limited by the synthesis process, which requires centrifugation to concentrate the precipitated antibodies and to produce the microparticles (**Figure** **1**a). Centrifugation, as a batch process, is not scalable, and it is inefficient for concentrating the aqueous suspensions, which are the precursor to the hydrogel microparticles. In addition, the particles generated through centrifugal dripping are limited to >100 µm in diameter, while smaller particles may be desired for ease of injection or for different therapeutic applications.

Solid protein microparticles have also been formed through solvent‐based dehydration. The seminal example is the Microglassification technique developed by Needham and colleagues, where protein microdroplets were dehydrated in an organic solvent to form ultra‐high concentration solid microbeads (Figure 1b).^[^ ^14^, ^15^, ^16^ ^]^ Further work showed that this technique was a viable alternative to lyophilization to form solid proteins that could be reconstituted at low concentrations in solution, or delivered at high concentrations with a non‐aqueous carrier.^[^ ^17^, ^18^ ^]^ Other groups used similar solvent‐based dehydration techniques to make solid protein microparticles with improvements to particle polydispersity and process scalability, which were both issues highlighted in the prior works.^[^ ^19^, ^20^ ^]^ However, none of these aforementioned formulations were suitable for aqueous reconstitution at high concentrations. These solid proteins are not stable in aqueous solution and would redissolve, resulting again in the issues that high‐concentration protein solutions face.

The aim of this work is to develop a new formulation process for highly concentrated (>350 mg mL^−1^) antibodies, which can be encapsulated into hydrogel microparticles and produced in a continuous fashion. The final formulation consists of antibody amorphous solid dispersion (ASD)‐laden alginate microparticles suspended in an aqueous PEG solution, where the solid antibody is uniquely stable in aqueous suspension at high concentrations. The antibody is precipitated into an ASD in situ through a solvent‐based dehydration process, where antibody‐PEG droplets are emulsified and dehydrated in pentanol, thus becoming concentrated into high‐concentration ASD‐containing microparticles (Figure 1c). The droplets are initially a stable single phase but upon dehydration the PEG and protein concentrations are driven into a region of their phase diagram where ASD formation spontaneously occurs. Alginate can be added to the dispersed phased and calcium to the continuous phase to trap the ASD inside a hydrogel matrix. We leverage the ease and scalability of microfluidic processing to yield hydrogel particles with tight control over a broad range of accessible particle sizes. The process results in an aqueous hydrogel microparticle formulation with desirable flow properties at high antibody concentrations. We envision this formulation platform to be generalizable to multiple therapeutic modalities and dosage or encapsulation forms.

## 2 Results and Discussion

### 2.1 Antibody Phase Behavior

Given that the dehydration process in our proposed formulation scheme requires ASD precipitation, the phase behavior of human IgG was studied as a function of PEG concentration, at ambient temperature. In this work, IgG was chosen as a model antibody drug as most clinically approved antibodies are IgG types.^[^ ^21^ ^]^ PEG is commonly used to induce phase separation of proteins, where excluded‐volume effects result in attractive depletion interactions that cause the proteins to aggregate and form a condensed phase.^[^ ^22^, ^23^ ^]^ The exact phase that is formed can depend on several factors, including PEG and protein concentrations, pH, and ionic strength. However, in most PEG‐induced precipitation studies, the different types of precipitates are often not distinguished from one another as there may only be one phase transition of interest, whether it is liquid–liquid phase separation (LLPS) for determining protein solubility, or crystallization for protein purification.^[^ ^24^, ^25^ ^]^ However, for the formulation of solid proteins, distinguishing liquid–liquid and liquid–solid phase separation is key, due to colloidal instabilities in the protein's liquid phases.^[^ ^26^ ^]^ Because solid proteins are typically in the form of dry powders and precipitation is generally not desired for protein formulations, PEG‐induced precipitation has not yet been well‐studied in the context of solid protein formulation.

**Figure 2:**
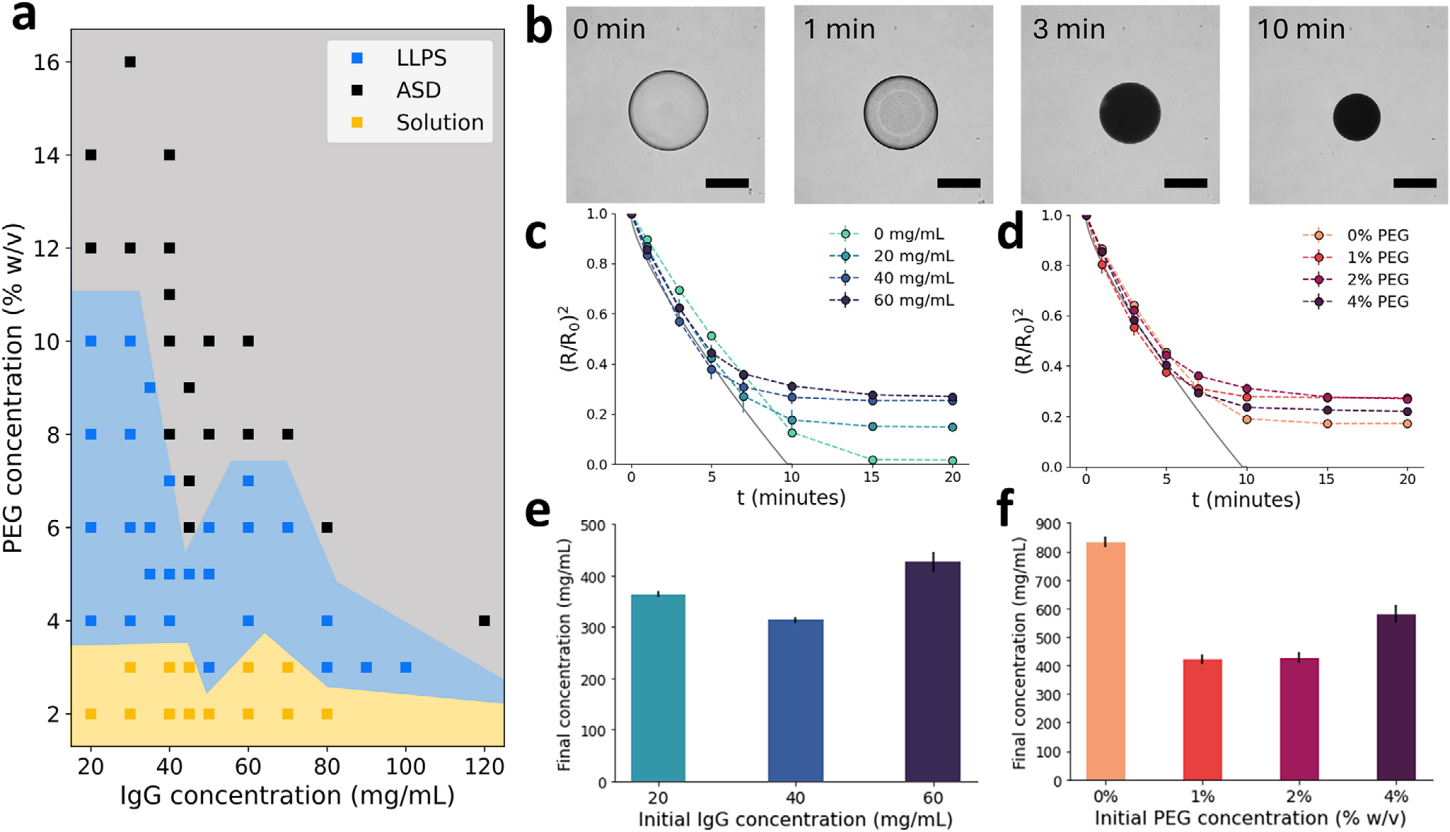
a) Phase diagram for IgG with varying PEG and IgG concentration, with discrete points correlating to experimental conditions and shaded areas corresponding to the estimated phase boundaries. Three phases are depicted, solution (solubilized IgG), LLPS (liquid–liquid phase separation), and ASD (amorphous solid dispersion). b) Time‐lapse images of 60 mg mL^−1^ IgG, 2% PEG in pentanol (0.4% w/v Tween 80). Scale bar = 200 µm. Change in scaled radius over time (min) for microparticles with varying concentrations of c) IgG and d) PEG in pentanol (0.4% w/v Tween80). The kinetic data for radius is plotted with the Epstein–Plesset model (solid grey line) for a pure water droplet of a comparable size, from ref. [27]. Final IgG concentrations (mg mL^−1^), estimated from the initial droplet IgG concentration and the calculated concentration factor of the droplets, shown for varying e) initial droplet IgG concentration (mg mL^−1^), and f) initial droplet PEG concentration (% w/v). *n*  =  3 for each condition.

Here, we present the phase diagram for IgG at various PEG and protein concentrations, shown in **Figure** **2**a. Buffer conditions were consistent with the desired formulation. A solubility curve for IgG was constructed at various PEG concentrations (Figure S1, Supporting Information). Details distinguishing the LLPS and amorphous solid dispersion (ASD) precipitates are also available in the Supporting Information (Figure S2, Supporting Information). In this case, ASD refers to the suspension of amorphous solid antibodies dispersed in the PEG carrier. At low PEG concentrations (⩽3% w/v), all mixtures were stable in the liquid phase regardless of the antibody concentration (⩽80 mg mL^−1^). Higher PEG concentrations induced the formation of ASDs preferentially over LLPS, with a viscous liquid condensate formed at moderate PEG concentrations and a white solid precipitate formed at high PEG concentrations, consistent with previous reports.^[^ ^22^, ^28^ ^]^ The exact nature of the phase transition was also dependent on the protein concentration. For IgG concentrations above 40 mg mL^−1^, 8% w/v PEG was sufficient to induce the formation of an ASD. This phase transition is reversible, as IgG will resolubilize when the PEG concentration is sufficiently low.^[^ ^25^, ^29^ ^]^ In the proposed formulation scheme, both PEG and IgG concentration within the microdroplet would increase upon dehydration in the outer organic phase, first forming liquid–liquid separated phases and then inducing precipitation of IgG in situ into an amorphous solid microparticle.

### 2.2 Single Microparticle Dehydration

To establish the microparticle formulation scheme, this solvent‐based dehydration process was studied on the single microparticle‐level. Validation and optimization of the experimental setup is discussed in the Supporting Information (Figure S3, Supporting Information). Figure 2b shows time‐lapsed images of a single antibody microdroplet in a pentanol bath under brightfield microscopy. The microdroplet initially contains IgG and PEG, with the concentration of its components increasing as solvent‐based dehydration occurs. The initial buffer concentration (5 mM) in the droplet was kept to a minimum to reduce the total salt content in the final particle. The microparticle remains spherical throughout the dehydration process due to the presence of surfactants in the surrounding organic solvent to control interfacial tension (Figure S4a, Supporting Information). We can qualitatively observe the antibody's phase transitions from changes in the droplet's opacity, corresponding with the previously discussed phase behavior. Concentrations of PEG and IgG within the microparticle can also be estimated by the measured change in droplet size. At *t* = 0 min, IgG (60 mg mL^−1^) is soluble at a low PEG concentration (2% w/v), as observed by the clear droplet. At *t*  =  1 min, liquid–liquid phase separation is observed from the cloudy droplet due to the second liquid phase as water is extracted. By *t*  =  3 min, increasing PEG concentration (∼4% w/v) within the droplet induces precipitation of the IgG (∼120 mg mL^−1^) into the amorphous solid form, resulting in an opaque particle. This solid–liquid transition is primarily driven by the increasing concentration of PEG, which quickly induces precipitation of the antibody. By contrast, without PEG in the initial droplet, the antibody remains in solution until reaching supersaturation and nucleating into an amorphous solid, a process that occurs on a much longer timescale than PEG‐induced precipitation due to the generally high solubility of IgG (Figure S4b, Supporting Information).^[^ ^30^ ^]^ With PEG present in the initial droplet, the antibody is incorporated into an amorphous solid dispersion, which will remain solid at sufficient PEG concentrations.

To investigate the kinetics of the dehydration process for various initial conditions, the relative change in droplet radius was recorded over time for varying initial IgG and PEG concentration, shown in Figure 2c,d, respectively. When normalized by surface area ($R_{0}^{2}$), the kinetics of the dehydration process were not affected by changes in solute concentration. In the initial time period, the kinetic data fit well to the Epstein–Plesset (EP) model before the particle plateaued to its final equilibrium size.^[^ ^27^ ^]^ As expected, the normalized equilibrium size of the particle was dependent on the initial concentrations of each solute, but interestingly did not show a directly proportional relationship. In fact, droplets with 40 and 60 mg mL^−1^ inital IgG concentration (C_*IgG*, *o*_) resulted in particles of similar size despite the difference in solids content, suggesting that packing of the amorphous antibody was more efficient in the case of C_*IgG*, *o*_  =  60 mg mL^−1^. This effect is highlighted when the final concentration of IgG (C_*IgG*, *f*_) in the particle is estimated using the change in droplet size. As shown in Figure 2e, C_*IgG*, *f*_ is much higher for C_*IgG*, *o*_  =  60 mg mL^−1^ than the other conditions. Similarly, droplets initially with 2% and 4% w/v PEG displayed more efficient ASD packing compared to the C_*PEG*, *o*_ = 1% w/v condition, although we note that IgG solutions with 4% w/v PEG are not stable since the higher PEG concentration will lead to LLPS. The differences in ASD packing density based on the initial droplet composition could be due to increased attractive interactions in the cases of higher PEG or IgG concentration.^[^ ^31^ ^]^ These results suggest there are ideal conditions to achieve the highest ASD packing density and thus concentration, which could be a strategy for optimizing ultra‐high concentration solid antibody formulations.

Without PEG in the initial droplet (C_*PEG*, *o*_ = 0% w/v), the change in size and thus concentration was the largest due to the absence of a polymer matrix. We can estimate C_*IgG*, *f*_ within these formed particles to be ≈830 mg mL^−1^, which is in the range of what Needham and colleagues had reported for various proteins.^[^ ^16^ ^]^ With the addition of PEG, the final particle concentrations were estimated to be between ≈300 and 600 mg mL^−1^ (Figure 2e,f). We expect these concentrations to be lower when PEG is initially present in the droplet, due to the higher total solids content and the retention of water in the polymer matrix of the ASD. As our results demonstrate, ASD formation is sensitive to both PEG and IgG concentrations within this range. As the final particle loading is dependent on the initial concentrations of each constituent, it is important to consider the formulation of the droplet solution. Regardless, the estimated C_*IgG*, *f*_ of these particles were still higher than what could be achieved with the previous process. Solvent‐based dehydration overcame the previous limitations and enabled ultra‐high concentration ASD‐laden particles.

**Figure 3:**
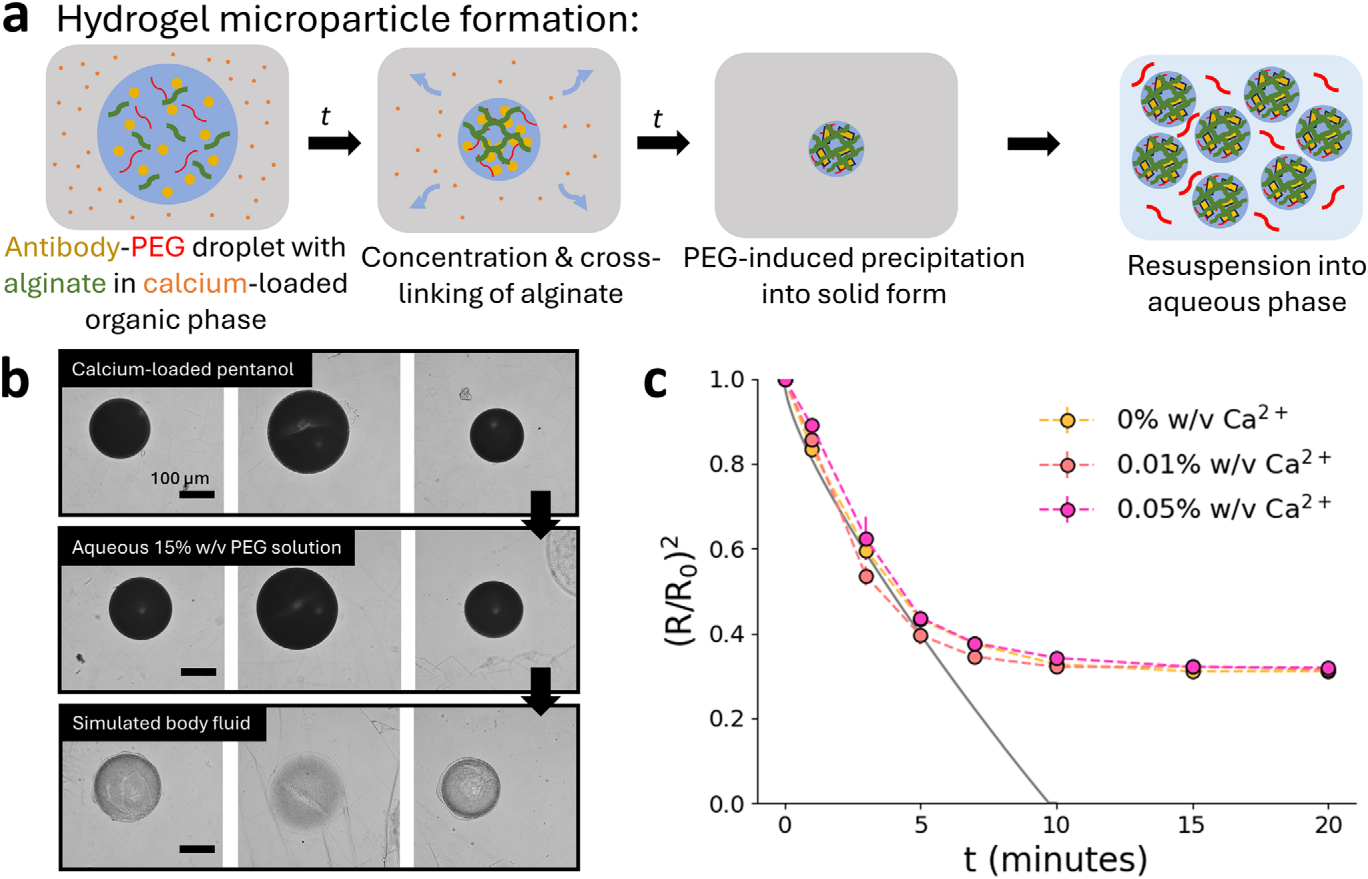
a) ASD‐laden hydrogel microparticle formation process. A droplet of antibody solution with precipitant (PEG) and polymer (alginate) is emulsified in an organic outer phase, extracting water from the droplet and concentrating both the antibody and PEG, which induces precipitation of the antibody into an ASD. Cross‐linker (calcium) in the organic phase cross‐links alginate simultaneously to dehydration and precipitation, resulting in a highly concentrated ASD‐laden hydrogel microparticle. The ASD is stabilized by PEG and the resulting microparticles can be resuspended in an aqueous phase with PEG for delivery, with hydrogel encapsulation improving the stability and injectability of the formulation. b) Images of individual ASD‐laden alginate microparticles demonstrating resuspension of the particles in PEG solution after dehydration, and dissolution of the ASD after release in simulated body fluid. c) Change in scaled radius over time (min) for hydrogel microparticles with various calcium concentration in pentanol (0.4% w/v Tween80). The kinetic data for radius is plotted with the Epstein–Plesset model (solid gray line) for a pure water droplet of a comparable size, from ref. [27].

While highly concentrated solid ASD microparticles were formed via this dehydration process, the resulting particles were brittle and not mechanically robust. In addition, we are aware from previous works that encapsulating antibody ASDs into hydrogel particles facilitates the stability and injectability of the formulation, due to the hydrogel masking protein–protein interactions and lending its lubricious rheological properties to the formulation.^[^ ^13^, ^32^ ^]^ Therefore, we sought to encapsulate the antibody ASD into hydrogel microparticles, concurrent to the dehydration process. To make cross‐linked hydrogel microparticles, alginate polymer (0.2% w/v) was dissolved in the antibody solution, and calcium chloride was dissolved in the outer pentanol phase. Alginate cross‐links ionically via association with calcium cations, leading to the formation of an alginate scaffold which encapsulates the solid antibodies as dehydration and protein precipitation occur (**Figure** **3**a).^[^ ^33^ ^]^ As proposed, these alginate microparticles can be resuspended into an aqueous solution with PEG to stabilize the solid antibody within the hydrogel. We first show this process for individual alginate microparticles loaded with IgG (Figure 3b). First, the droplets are simultaneously precipitated and cross‐linked in a pentanol bath, forming a opaque, concentrated solid microparticle. Then, the particles are transferred to an aqueous solution with 15% w/v PEG (buffered with 5 mM HEPES, pH 7.4). IgG remains encapsulated in the microparticle in its solid form as PEG will prevent dissolution of the antibody. We also show that this stabilization mechanism is due to PEG and does not depend upon the particle's alginate content (Figure S5, Supporting Information). Although including PEG in the initial droplet solution limited the particle loading, it enabled the formation of a stable ASD, which allowed for aqueous reconstitution of the particles. Finally, these particles are transferred into a simulated body fluid (SBF), where the drop in local PEG concentration will cause IgG to dissolve and diffuse from the particle, leaving behind the alginate scaffold. Without a sufficient PEG concentration, IgG will resolubilize, thus resulting in the blank hydrogel particles shown in Figure 3b. The antibody's phase behavior here demonstrates that the antibody can be viably delivered in its solid form in an aqueous vehicle and that its precipitation is reversible.

We also investigated the kinetics of the dehydration process for the alginate hydrogel microparticles. For droplets containing alginate, there was a slight increase in the normalized equilibrium particle size compared to those droplets with only IgG and PEG, owing to the additional polymer content in the particle (Figure 3c). However, the concentration of calcium cross‐linker in the pentanol bath did not affect either the kinetics or the equilibrium behavior of the droplets, with a final estimated concentration factor ($R_{o}^{3}/R_{f}^{3}$) of ≈6× for all conditions (Figure S6, Supporting Information). The cross‐linking of alginate forms a solid hydrogel network, which does not contribute to volume exclusion of the protein, so the cross‐linked alginate would ultimately not affect the antibody's phase behavior. These results showed that ASD‐laden hydrogel particles could reliably be produced and resuspended in various aqueous media with predictable behavior, which is crucial for its potential clinical applications.

### 2.3 Continuous Microparticle Encapsulation

**Figure 4:**
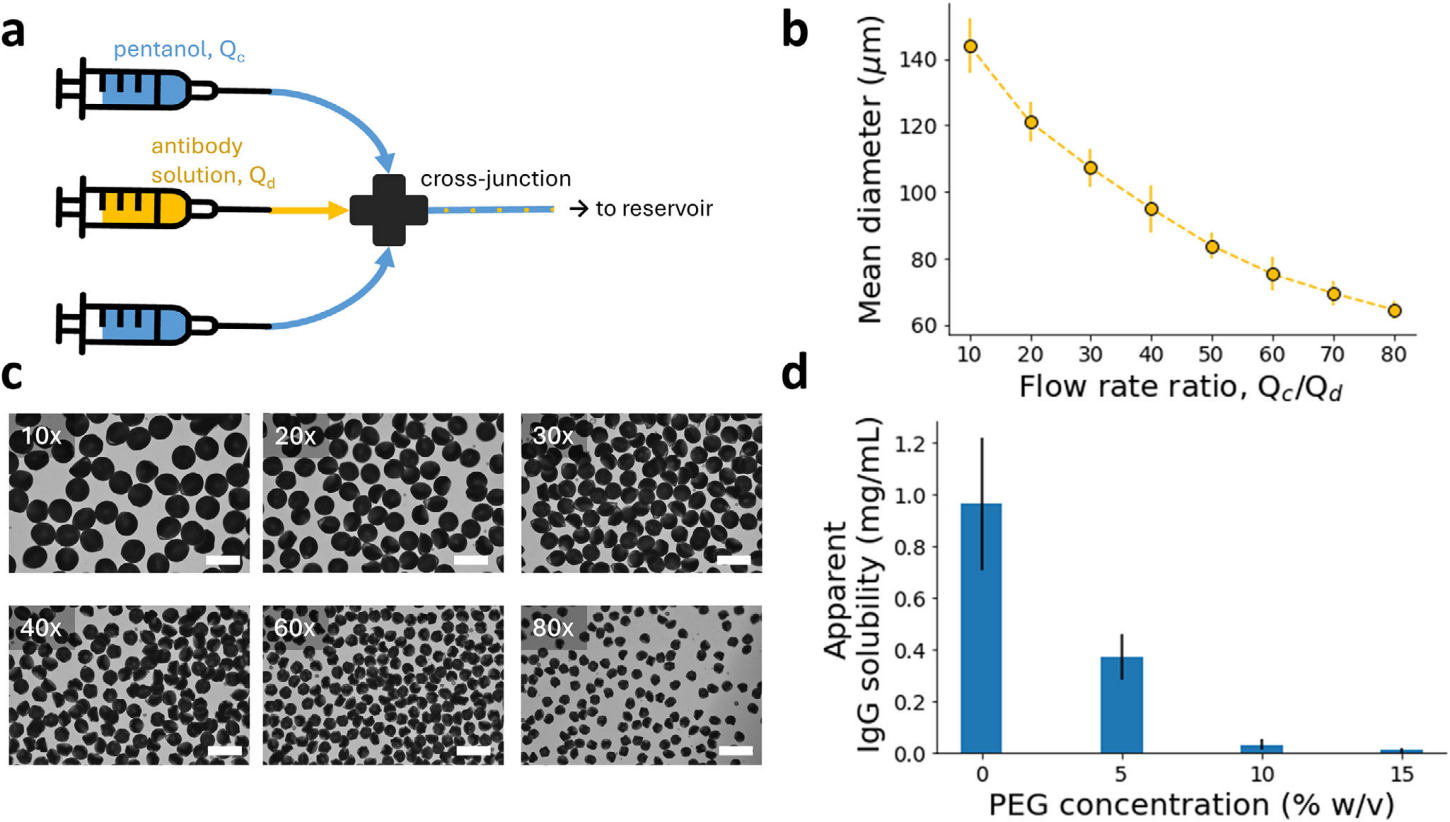
a) Schematic of the microfluidic cross‐junction used for microparticle generation. The continuous phase, pentanol is fed into two inlets perpendicular to the antibody solution, causing pinch‐off of the dispersed (antibody) phase, where the antibody droplets are dried in the continuous stream and fed into a reservoir for particle collection. b) Mean particle diameter (µm) versus flow rate ratio for the microparticle generation process. c) Representative images of ASD‐laden hydrogel particles produced at various flow rate ratios in the microfluidic process. Scale bar = 200 µm. d) Apparent solubility of IgG (mg mL^−1^) from the hydrogel particles in aqueous solutions of varying PEG concentration (% w/v), based on a total concentration of 1 mg mL^−1^ IgG.

For application of the formulation, we sought to produce the ASD‐laden hydrogel particles in a scalable manner. To demonstrate scalability of this solvent‐based dehydration process for solid mAb formulation, continuous encapsulation of antibody into alginate microparticles via solvent‐based dehydration was accomplished via a microfluidic cross‐junction (**Figure** **4**a). IgG solution containing PEG and alginate was dispersed in a continuous pentanol phase, with the dispersed droplets precipitating and cross‐linking into hydrogel microparticles. Due to the immiscibility of the two fluid phases, antibody droplets were easily formed via pinch‐off in the continuously flowing pentanol phase.^[^ ^34^ ^]^ Surfactant (Tween 80) was used in the continuous phase to control interfacial tension. The flow rate of the dispersed phase was held constant at *Q*_*d*_ = 4 µL min^−1^ while the flow rate of the continuous phase (*Q*_*c*_) was varied to influence the hydrogel particle size (Figure 4b). As the flow rate ratio *Q*_*c*_/*Q*_*d*_ increased, smaller particles were produced due to the greater shear force of the continuous phase at the cross‐junction. The size of the cross‐junction thru‐hole (150 µm) also controlled the range of particle sizes that were achieved. Figure 4c shows representative images of antibody‐laden hydrogel microparticles produced at various flow rate ratios, with generally consistent particle size and sphericity. For *Q*_*c*_/*Q*_*d*_ >40, particles <100 µm in diameter could be generated. This size range was previously inaccessible in our prior work based on limitations of centrifugal synthesis.^[^ ^12^, ^13^ ^]^ The ability to generate particles in this size range represents a significant advance over the previous process, especially for subcutaneous injection where thinner needles are desired. However, the greater shear forces at higher flow rate ratios also resulted in the fragmentation and formation of satellite particles. As such, a moderate flow rate ratio (30x) was employed to produce particles for analysis, owing to the more uniform particle morphology and size distribution at this ratio. Previous encapsulation processes for ASD‐laden hydrogel particles relied on centrifugation, which was neither continuous nor scalable. This encapsulation process was able to continuously generate microparticles with a wide range of characteristic diameters suitable for injection and moderately tight control over particle size. The droplet‐based microfluidic setup used for this process can be easily scaled up for high‐throughput applications.^[^ ^35^ ^]^ More advanced microfluidic designs could be utilized to increase output and meet other particle size specifications.

The continuous microfluidic encapsulation process was used to produce antibody ASD‐laden microparticles for further characterization. Particles of 105±16 µm diameter were used for characterization of particle loading and the further analysis described later in Section 2.4, as this particle size is representative of the range which can be feasibly produced by the current production method. The selected particle size for analysis was also in the range of clinically relevant particle sizes, between the macrophage capacity limit (20 µm) and the diameter of most subcutaneous needles (200 µm). Particles of different sizes within this operational range could potentially show differences regarding the protein loading or ASD stability, due to the influence of dehydration kinetics which was discussed in the previous section. For the purposes of complete formulation characterization, the particle size was controlled for one relevant size distribution (105±16 µm), but future studies would consider the effect of particle size distribution on formulation performance and characteristics to expand the generality of the formulation platform.

To determine the final formulation concentration, particle loading was measured by determining the sample volume of antibody‐laden particles and eluting antibody from the particles to measure total IgG mass in the particles. Microparticle loading is dependent on the initial IgG solution concentration (Figure 2e). For these particles with initial conditions *C*_*IgG*, *o*_ = 60 mg mL^−1^ and *C*_*PEG*, *o*_ = 2% w/v, the particle loading was calculated to be 486 ± 35 mg mL^−1^ (*n*  =  3), an ∼8x increase in concentration, which is a similar concentration factor to what was predicted in the single microparticle experiments. These initial concentrations of IgG and PEG were selected for the most efficient ASD packing with an initially stable IgG solution, based on the phase behavior and single‐particle experiments. The final particle loading was much higher than what could be achieved with our previous processes, which relied on centrifugation to concentrate and encapsulate the ASD.^[^ ^12^, ^13^ ^]^ The current process enabled continuous particle production and resulted in higher particle loadings, which represent significant improvements toward a platform for SC formulations. Initial droplet compositions could potentially be optimized to reach higher microparticle loadings, which would involve further studies on ideal conditions for ASD formation and packing. However, the current particle loading approaches the theoretical limit suggested by Garidel et al. (500 mg mL^−1^), indicating that packing efficiency within the particle is already quite high.^[^ ^36^ ^]^ Encapsulation efficiency (E.E.) of the process was defined as the mass of encapsulated antibody over the total mass of antibody in solution. To measure the E.E., the amount of IgG in pentanol bath after particle generation compared to the amount of IgG in the initial solution. The E.E. for the continuously generated microparticles was high (98.8 ± 0.2%), due to minimal leaching of the antibody from the droplets. We expect minimal leaching of antibody to occur during the dehydration process as the antibody is not soluble in pentanol. This E.E. is comparable to that of prior processes for antibody‐laden alginate microparticles, and is much higher than those of typical protein encapsulation processes.^[^ ^13^, ^32^ ^]^

A crucial limitation of previous formulation approaches for solid antibody formulations is the inability to resuspend these solids in aqueous phases, either requiring reconstitution in solution at low concentrations or delivery in non‐aqueous vehicles, which pose regulatory and manufacturing challenges. After continuously generating antibody‐laden hydrogel microparticles, we demonstrated that these particles are easily resuspendable and stable in aqueous solution, facilitating delivery in an aqueous vehicle. Due to the formation of the amorphous solid dispersion via PEG‐induced precipitation, the antibody is retained in its solid form even in aqueous solution as long as PEG is present in sufficient concentrations. We qualitatively showed this effect earlier for antibody‐laden alginate microparticles (Figure 3b). The apparent solubility of IgG from the particles in aqueous solutions with varying PEG concentration is shown in Figure 4d. Clearly, without PEG in solution (0% w/v), the antibody has a tendency to diffuse from the particle as previously discussed, resulting in a relatively high apparent IgG solubility. However, antibody solubility steadily decreases as the concentration of PEG in solution increases, which is expected as PEG is known to systematically decrease protein solubility.^[^ ^37^ ^]^ In fact, apparent IgG solubility in 10% and 15% w/v PEG solution is very low, 0.03 and 0.01 mg mL^−1^, respectively. Therefore, a 10% w/v PEG solution is sufficient to stabilize IgG in its solid form and prevent dissolution of antibody from the particles. This result is consistent with the phase diagram presented earlier (Figure 2a), where 10% w/v PEG induced and stabilized ASD formation at all protein concentrations. In this manner, the antibody‐laden particles can be delivered as an aqueous suspension rather than using non‐aqueous carriers as other high‐concentration formulations. The ability to deliver high‐concentration antibodies in aqueous suspension offers a key advance over the state‐of‐the‐art. Non‐aqueous suspensions lead to increased injection site pain, toxicity, and pose manufacturing complications and a regulatory burden that our approach overcomes.^[^ ^11^ ^]^

### 2.4 Applicability as a Formulation Platform

Further characterization of the antibody‐laden microparticles was carried out to support the applicability of the formulation process as a potential platform for subcutaneous delivery of antibodies. First, injection force tests were performed on the final aqueous particle suspension to assess injectability of the formulation. Compared to other material properties such as viscosity and storage and loss modulus, injection force is the most relevant measurement for clinical applications and yields interpretable quantitative results.^[^ ^38^ ^]^ Injectability is typically a challenge for SC delivery due to the viscosity of high‐concentration antibody solutions. Lower injection forces are desired for viability and ease of administration.^[^ ^39^ ^]^

**Figure 5:**
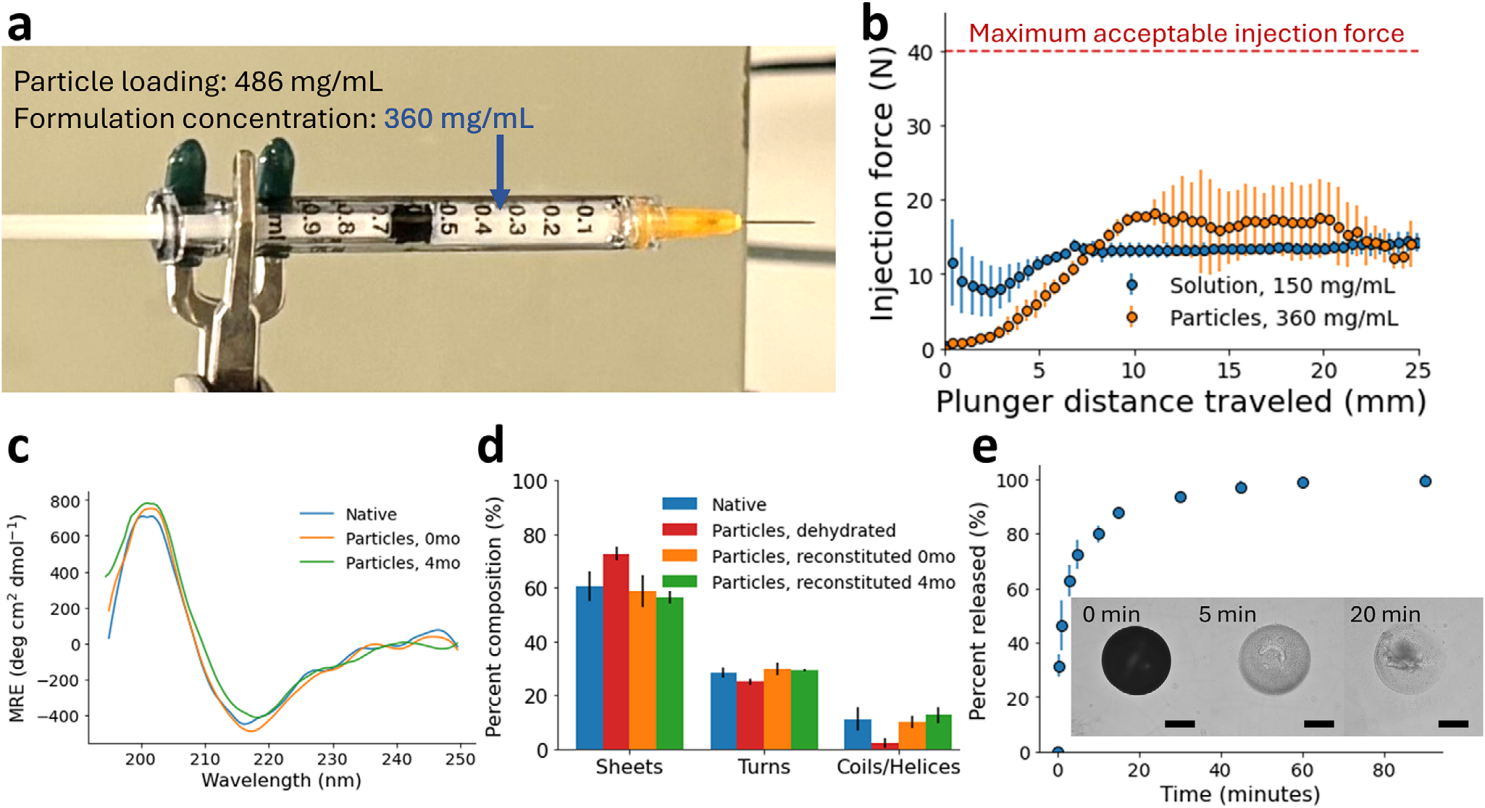
a) Digital camera image of a syringe loaded with the microparticle formulation at a formulation concentration of 360±9 mg mL^−1^. b) Injection force (N) versus plunger distance traveled (mm) for an IgG solution control (blue) and the microparticle formulation (orange) (*n*  =  3). Maximum injection force is shown for ref. [40]. c) Mean residual ellipticity (deg cm^2^ dmol^−1^) from circular dichroism measurements of native IgG reference (blue) and IgG reconstituted from hydrogel microparticles immediately after formulation (orange) and after 4 months of storage (green). d) Secondary structure composition as calculated from FTIR measurements for native IgG reference (blue), dehydrated IgG particles (red), and IgG reconstituted immediately after formulation (orange) and after 4 months of storage (green). e) Release profile over time for IgG released from hydrogel microparticles in simulated body fluid (SBF) at 37^*o*^C. Time‐lapse images of an ASD‐laden hydrogel microparticle in SBF are inset (scale bar = 50 µm).

The tests were performed using an universal mechanical tester to apply a downward compressive force on the syringe plunger over a specified displacement. A particle suspension with a formulation concentration (*C*_*form*_) of 360±9 mg mL^−1^ IgG was used, depicted in **Figure** **5**a. The *C*_*form*_ is based on the previously calculated particle loading and a particle volume fraction (*V*_*particles*_/*V*_*particles* + *PEGsolution*_) of 0.75 in 10% w/v PEG solution. The *C_form_* was confirmed by dissolving the formulation in phosphate‐buffered saline after performing the injection tests and measuring the supernatant concentration via UV–vis. This *C*_*form*_ represents a new upper limit in ultra‐high concentration aqueous antibody formulations, where previous formulations were limited to ⩽200–300 mg mL^−1^, due to the new dehydration process which overcame the concentration limitations of the previous processes. The increase in *C*_*form*_ is significant as higher concentrations enable lower volume doses or less frequent injections, both of which are favorable for accessibility and patient compliance.^[^ ^2^, ^41^ ^]^ The formulation was injected through a 25‐gauge needle, which is standard for subcutaneous injection.^[^ ^42^ ^]^ A flow rate of 25 µL s^−1^ was selected for the tests as a moderate, clinically relevant injection speed.^[^ ^43^ ^]^ Figure 5b shows the average injection force profile over the syringe plunger displacement. In the beginning of the test, the microparticle formulation experiences a “start‐up” regime where the injection force increases until it reaches a plateau, which is typical for injection force tests. In the plateau regime, there are still variations in the injection force profile as the plunger moves, both within and across individual tests. This suggests that there are local differences in the distribution of the particles and the injection force may experience some dependence on how the formulation is loaded.^[^ ^38^ ^]^ The force profile in this plateau regime can be averaged to yield the glide force, which ranged between 10 and 20 N for the tests. These glide forces were comparable to those reported for non‐aqueous mAb suspensions at similar antibody concentrations.^[^ ^44^ ^]^ Regardless of the variations, the injection force profiles for all tests were well‐below the suggested limit of 40 N as the maximum acceptable force for subcutaneous injection.^[^ ^40^ ^]^ Also, the same injection tests for an IgG solution (150±4 mg mL^−1^) as a control resulted in comparable glide forces as the microparticle formulation, even though the microparticle formulation was >2 times more concentrated than the solution. A stable IgG solution at a higher concentration was not possible for us to make. The relatively low injection forces of the microparticle formulation can be attributed to the encapsulation of the antibodies into hydrogels, which lend advantageous rheological properties, such as particle deformability and shear‐thinning behavior and help to improve injectability.^[^ ^32^ ^]^ Ease of injection is key for SC delivery to enable self‐administration and home‐based care which are touted as major benefits of SC delivery.^[^ ^1^, ^45^ ^]^ These results show that the particle formulations can be injected at ultra‐high concentrations with well‐accepted injection forces. Thus, our formulation is viable for self‐administration, which improves accessibility, patient compliance, and quality‐of‐life.^[^ ^2^ ^]^

To show the structural stability of the antibody after dehydration and encapsulation, characterization of the antibody secondary structure was performed via both far‐UV circular dichroism (CD) and FTIR‐ATR spectroscopy. Following microparticle generation immediately and after 4 months of storage at 4° C in 10% w/v PEG solution, the antibody was eluted from the particles into solution for analysis. The far‐UV CD data (Figure 5c) showed comparable mean residual ellipiticity between native IgG reference and the reconstituted IgG from the microparticles, indicating that the structure and conformation of the antibody was not irreversibly affected by the dehydration‐encapsulation process. FTIR spectroscopy was used to quantify the secondary structural composition, via deconvolution and analysis of the amide I band region (1700–1600 cm^−1^). The estimated compositions of the native, dehydrated (prior to reconstitution), and reconstituted IgG are shown in Figure 5d. Representative FTIR spectra are available in the Supporting Information (Figure S7). Both the native and reconstituted IgG conditions were consisted of majority β‐sheet structure (≈60%), which is consistent with IgG antibody literature.^[^ ^46^, ^47^, ^48^ ^]^ The percent composition of β‐turns (≈30%) and α‐helices or random coils (≈10%) were also in the range of previous reports, and there were no significant differences between the native reference and reconstituted antibody composition. Dehydration is known to perturb antibody structure due to the removal of water and rearrangement of intermolecular interactions, displayed in the composition of the dehydrated particles having significantly more β‐sheet structures (≈75%), which is typical of dehydration‐induced aggregation.^[^ ^49^, ^50^ ^]^ However, these effects were shown to be reversible for the microparticle formulation after dissolution of the antibody in aqueous media, with the reconstituted IgG displaying its native structure. These results were similar to those observed in the Microglassification^TM^ technique, where drying‐induced structural changes were reversed upon dissolution.^[^ ^14^, ^16^ ^]^ For our formulation platform, PEG‐induced precipitation of the antibody during the dehydration process resulted in a stable ASD, where PEG preserved the solid antibody form, given that the antibody's native structure was recovered upon dissolution.

In addition, this structure was stable over 4 months of storage in the microparticle suspension, as the antibody released at 4 months was not significantly different from either the native or the antibody released immediately after generation. Moreover, ELISA bioactivity assay was performed, also for IgG samples released following formulation and after 4 months of storage in the microparticle form. The relative bioactivity for both samples were 100%, showing that the formulation process and resulting dosage form are compatible with the structural and functional stability of the antibody (Figure S8, Supporting Information). The long‐term stability of the antibody in this formulation is a key result, showing that the antibody is stable in the microparticle suspension over several months, which has important implications for the formulation's applicability in delivery and treatment. Size exclusion chromatography (SEC) was also performed to confirm that irreversible aggregation had not occurred during the dehydration process, with >90% of the antibody recovered from the particle as monomers (Figure S9, Supporting Information). Although outside the scope of the current work, future work would consider in vivo bioavailability and immunogenicity studies to further the clinical applicability of the platform. Similar formulations reported in the literature with associated in vivo studies, such as crystalline antibody‐laden alginate particles, mAb‐laden chitosan hydrogel, and non‐aqueous solid mAb particle suspension, have shown predictable pharmacokinetics and no observed adverse effects.^[^ ^12^, ^51^, ^52^ ^]^ The robust in vitro characterization in this work along with the successful in vivo performance of other similar formulations supports the potential for our proposed platform to be viable for clinical applications.

Finally, we demonstrated that the antibody could be easily released from the particle suspension upon injection in a physiological media. Release of the antibody from the alginate microparticle suspension was studied in vitro at physiological conditions using simulated body fluid (SBF) as the release media. The release profile for IgG at 37 ^°^C is shown in Figure 5e. The release of the antibody from an individual microparticle submerged in SBF is also visualized by observing a decrease in the opacity of the particle over time. Upon injection into SBF, the microparticle suspension is dispersed, and the local PEG concentration in the microparticle decreases, which triggers dissolution of the antibody and diffusion from the particle, as described earlier. This process occurs rapidly, with most of the antibody (∼80%) released within the first 10 min of the test, which is comparable to other reports for high‐concentration solid antibody formulations.^[^ ^13^, ^53^ ^]^ Fast release of the antibody is common for hydrogel‐based delivery systems, due to the high permeability of water in the hydrogel. Altogether, characterization of the ASD‐laden microparticles indicates that the formulation has acceptable injectability, stability, and release properties, advancing this solvent‐based dehydration process as a viable platform to formulate high‐concentration solid antibodies.

## 3 Conclusion

Subcutaneous administration of therapeutic antibodies requires high‐concentration formulations for reduced injection frequency or volume and improved patient compliance and quality‐of‐life. In this work, we present for the first time an ultra‐high concentration (360 mg mL^−1^) aqueous antibody formulation with acceptable injectability and stability properties. Solvent‐based dehydration was applied to concentrate antibody droplets and trigger an in situ PEG‐induced phase transition from liquid to solid antibody, using IgG as a model drug. We continuously synthesized amorphous antibody‐laden hydrogel microparticles through a facile microfluidic process which can be modulated to achieve different output parameters such as particle size. The microparticles were suspended in PEG solution where the antibody was retained in its solid form, uniquely combining the stability of a solid antibody formulation with the benefits of an aqueous dosage form. Due to the advantages of hydrogels for therapeutic encapsulation and delivery, the final formulation was able to be injected at a high concentration, while showing rapid release in a simulated body fluid. Overall, the results of this work demonstrate the potential of our ASD microparticle formulation as a platform to formulate high‐concentration antibodies for SC delivery. In addition, we expect this process to be generalizable, both in terms of the therapeutic modalities and the dosage form. While we validated the process here for an amorphous antibody, other solid states (i.e., crystalline, coacervate) would be also suitable for formulation. It is viable for the platform to be expanded to other therapeutic molecules (i.e., mAbs, peptides, nucleic acids) as long as the molecule can be stabilized in its solid form, whether via PEG or another precipitant. The encapuslation process could also be modified with different polymers and cross‐linkers to design hydrogel particles with desired rheological properties or release profiles.

## 4 Experimental Section

### Materials

All chemicals used were of analytical grade. Lyophilized total human IgG was purchased from Equitech‐Bio, Inc. Poly(ethylene) glycol (PEG, 3350 kDa) was purchased from Rigaku Reagents. Sodium alginate (5–40 cP) was purchased from Sigma. All other chemicals were purchased from Sigma and used without further purification.

### Phase Transitions in IgG Solutions

To study PEG‐induced phase separation in IgG, concentrated PEG solution (50% w/v) was added dropwise to a stock IgG solution, prepared from the lyophilized product, while stirring. The mixtures were buffered at pH 7.4 with 5 mM HEPES (N‐2‐hydroxyethylpiperazine‐N‐2‐ethane sulfonic acid) and was carried out in batches at a total volume of 0.3 mL. All solutions were filtered with a 0.2 µm filter before mixing. The resulting mixture was kept at room temperature for 1 h while rotating at 20 rpm on a tube mixer. Afterward, the mixture was centrifuged at 1700 RCF for 30 min to recover any precipitates. To determine the solubility of IgG at various PEG concentrations, the protein concentration in the supernatant after centrifugation was measured in a UV–vis Nanodrop spectrophotometer using the 280 nm absorbance method. To distinguish between liquid–liquid and liquid–solid phase transitions, the appearance of the precipitates was evaluated.

### Single Microparticle Dehydration Experimental Setup

Single droplets of antibody solution (0–60 mg mL^−1^) containing PEG (0–4 w/v%) were expelled from a syringe with a 30G stainless steel needle (I.D. = 159 µm, O.D. = 312 µm) into a 20 mL 1‐pentanol (0.4% w/v Tween 80) reservoir. The pentanol reservoir was held in a glass‐bottom Petri dish spin‐coated with polystyrene to hydrophobize the bottom surface and minimize contact of the microdroplet with the dish. The Petri dish was mounted upon an inverted optical microscope equipped with a digital camera to record the dehydration of single microparticles. Frames from the captured video were analyzed using ImageJ to measure the droplet diameter during dehydration and the size of the dehydrated microparticle. All experiments were performed at ambient temperature with an initial microdroplet radius between 150 and 200 µm, with *n*  =  3 for each condition.

### Continuous Antibody Encapsulation

IgG ASD‐laden alginate microparticles were produced continuously using a microfluidic cross‐junction (IDEX PEEK P‐891, thru hole = 0.15 mm, O.D. = 1/16 in) with soft‐walled tubing (PTFE, I.D. = 0.3 mm, O.D. = 1/16 in). The cross‐junction consisted of a cross‐shaped channel intersection with four total inlet and outlet ports (Figure 4a). The dispersed phase containing aqueous antibody solution (40–60 mg mL^−1^) with PEG (1–4 w/v%), sodium alginate (0.2 w/v%), and 5 mM HEPES (pH 7.4) is introduced through one inlet, while the continuous phase consisting of 1‐pentanol with calcium chloride (0.01 wt%) and Tween 80 (0–0.4 wt%) is delivered through the two inlets perpendicular to the dispersed phase. The solutions are loaded into syringes and connected to separate syringe pumps (Harvard Apparatus PHD 2000). The flow rate of the dispersed input was controlled at 4 µL min^−1^ while the flow rate of each continuous input was varied between 40 and 320 µL min^−1^. To minimize pressure variations and ensure reproducible results, all tubing and the microcross are secured flat against a level surface. The microparticle samples were collected at steady state in a pentanol bath at the outlet port. Images of the microparticles were taken after complete dehydration under brightfield microscopy. The size distribution of the microparticles was analyzed for each condition using the StarDist object detection method to obtain final particle diameter (*n* > 30).^[^ ^54^, ^55^, ^56^ ^]^

### Antibody Particle Stability in PEG Solution

For evaluating the apparent solubility of the amorphous solid antibody in suspension with PEG to ensure the stability of the solid phase in the aqueous phase, IgG ASD‐laden alginate microparticles were generated via the microfluidic process described. The samples were transferred to a microcentrifuge tube and excess solvent was removed to adjust the total IgG content in each tube to 1 mg. 1 mL of storage buffer (5 mM HEPES, pH 7.4) with different w/v% concentrations of PEG was added each tube and the samples were left to equilibrate with the storage buffer at room temperature (∼22^°^C). After 24 h, the protein concentration in the supernatant was measured using the 280 nm absorbance method.

### Injection Force Measurements

The injectability of the microparticle formulation was quantified using injection force measurements according to a previously described protocol.^[^ ^32^ ^]^ A Zwick–Roell universal testing machine (model Z010) equipped with a 500 N load cell and custom 3D‐printed compression test flat‐plate attachment was used. All tests were performed at ambient conditions with multiple sample replicates (*n*  =  3). The particle suspension formulation was loaded into a BD 1‐mL syringe with a 25‐gauge (ID = 260 µm, OD = 515 µm) Luer‐lock needle. The syringe was clamped in place during the test. For each experiment, the stroke distance (displacement) was controlled at 25 mm, corresponding to a ∼0.5 mL injection volume. The stroke speed was set at ∼1.4 mm s^−1^, which corresponds to a injection rate of 25 µL s^−1^.^[^ ^57^ ^]^

### Circular Dichroism (CD)

Far‐UV circular dichroism (CD) was used for IgG secondary structure determination, using a JASCO J‐1500 spectropolarimeter. IgG samples were prepared at 0.5–1 mg mL^−1^ in aqueous solution (5 mM HEPES, pH 7.4), and spectra were obtained in a 1 mm quartz cuvette (Hellma Analytics) at ambient conditions. The wavelength was scanned from 250–190 nm with a 0.5 nm data pitch, 1 nm bandwith, 4 s digital integration time, and 50 nm min^−1^ scanning speed. A nitrogen gas purge was run for at least 5 min before sample measurement. The spectra recorded were the average of five individual background‐subtracted scans, and the data was reported as mean residual ellipticity following the protocol in Kelly et al.^[^ ^58^ ^]^

### Fourier Transform Infrared Spectroscopy (FTIR)

Solvated and solid state samples of IgG were analyzed using FTIR spectroscopy (Thermo Fisher Nicolet is50) with a built‐in diamond ATR crystal. Either a 20 µL drop of IgG sample, 40 mg mL^−1^ concentration in simulated body fluid (SBF) for solvated samples or a powder for solid state samples was placed on the ATR crystal. 256 scans were collected at a resolution of 4 cm^−1^, with the background spectrum automatically subtracted. The spectra were analyzed using Fityk software. The amide I band region (1700–1600 cm^−1^) was isolated, baseline‐corrected, and area‐normalized. The second derivative spectra was obtained using 9‐point Savitzky–Golay smoothing algorithm and peak‐fitted with a Gaussian curve function and the Levenberg–Marquardt algorithm. Peaks were assigned to secondary structures following the literature conventions.^[^ ^59^, ^60^ ^]^ The relative areas of the fitted peaks were used for the quantification of IgG secondary structure (*n*  =  5 for each sample).

### In Vitro Release Assays

To evaluate release of the antibody from the hydrogel microparticle, approximately 20 µL of the antibody‐laden microparticle aqueous suspension was injected into a 2 mL centrifuge tube filled with 1 mL of pre‐warmed (37^°^C) simulated body fluid (SBF), which was prepared based on the literature to mimic the ionic composition of the SC environment, comprised of 7.996 g L^−1^ sodium chloride, 0.350 g L^−1^ sodium bicarbonate, 0.224 g L^−1^ potassium chloride, 0.228 g L^−1^ potassium phosphate dibasic trihydrate, 0.305 g L^−1^ magnesium chloride hexahydrate, 0.278 g L^−1^ calcium chloride, 0.071 g L^−1^ sodium sulfate, 6.057 g L^−1^ tris (hydroxymethyl) aminomethane, and 40 mLL^−1^ of 1 M hydrochloric acid.^[^ ^61^ ^]^ At specified time intervals, 200 µL of the supernatant was removed and taken for measurement of protein concentration via the 280 nm UV–vis absorbance method, and the sampled volume was replaced with fresh SBF. The volume of the initial particle suspension for release was chosen such that the total antibody concentration in the release medium would be <10 mg mL^−1^, ensuring the medium would not be saturated during the test period. Measurements were taken in triplicate using multiple sample replicates (*n*  =  3).

## Conflict of Interest

Massachusetts Institute of Technology (MIT) has filed a provisional patent application on behalf of P.S.D., T.Z., and L.A. based on the research in this study.

## Supporting information

Supporting Information

## Data Availability

The data that support the findings of this study are available from the corresponding author upon reasonable request.
