# Supplementary material for: High‐Concentration Antibody Formulation via Solvent‐Based Dehydration
Source: Adv Mater. 2025 Nov 23;38(7):e16429. doi: 10.1002/adma.202516429 (PMC12862716; doi:10.1002/adma.202516429)
Supplement: Supplementary file 1 — Supporting Information [file ADMA-38-e16429-s001.pdf]

ADVANCED MATERIALS

# Supporting Information

for *Adv. Mater.*, DOI 10.1002/adma.202516429

High-Concentration Antibody Formulation via Solvent-Based Dehydration

*Talia Zheng, Lucas Attia, Janet Teng and Patrick S. Doyle**

# Supplementary Information: High-concentration antibody formulation via solvent-based dehydration

*Talia Zheng  Lucas Attia  Janet Teng  Patrick S. Doyle**

## IgG phase behavior and solubility

To determine the solubility of IgG, the concentration of IgG in the supernatant was measured using an UV-vis spectrophotometer for different precipitation conditions. The concentration of the supernatant was then taken as the apparent solubility of IgG, S. Figure S1 shows log(S) at varying PEG and IgG concentration, coded either by initial IgG concentration or the condensed phase. Regardless of the nature of the condensed phase or the initial protein concentration, IgG solubility exhibited characteristic linear dependence of log(S) on the PEG concentration [1]. Extrapolating from the linear fit, the apparent solubility of IgG at these conditions (5 mM HEPES, pH 7.4) for zero PEG concentration is ∼215 mg/mL (log(S) = 5.4). The log(S) value at zero PEG is within the range of other reported apparent solubilities for IgG-type antibodies, with variations based on pH and ionic strength [2]. It is clear though that for $C_{IgG} > 20$ mg/mL and $C_{PEG} > 8\%$ w/v, the condensed phase that formed was an amorphous solid rather than liquid phase.

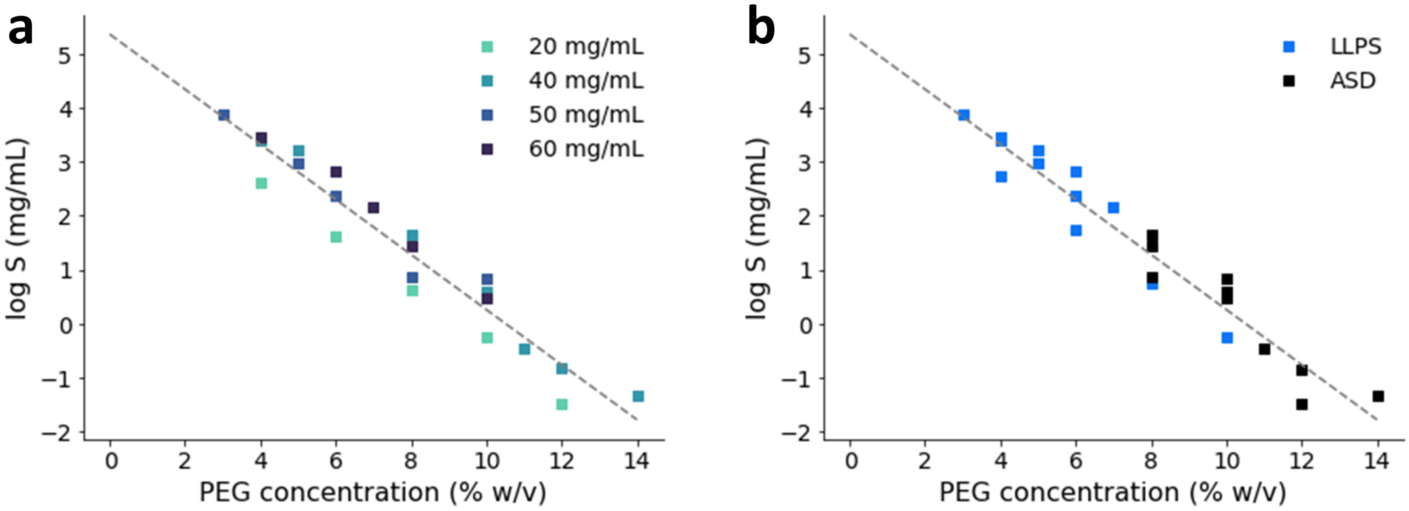


Figure S1: log S (solubility, mg/mL) for IgG and PEG mixtures with various initial conditions, based off the data in Figure 2a. Solubility data points are differentiated by (a) initial IgG concentration and (b) resulting phase transition.

In addition, Figure S2 shows qualitatively the differences observed between LLPS and ASD cases. LLPS was observed as dispersed droplets in a second liquid phase under microscopy and as a clear or translucent precipitate, whereas ASD was observed as fractal-like solid protein aggregates under microscopy and as a opaque white precipitate.

## Single droplet experimental setup validation

The Epstein-Plesset equation was first developed in 1950 for the dissolution rate of a gas bubble in liquid-gas solution:

$$\frac{dR}{dt} = -\alpha \left\{ \frac{1}{R} + \frac{1}{\sqrt{\pi D t}} \right\} \tag{1}$$

where $\alpha = Dc_s/\rho(1-f)$, $f = c_i/c_s$, and $c_i$, $c_s$ being the initial and saturated concentration in the bulk phase. The initial saturation ratio of pentanol, $f$, was assumed to be 0 for these experiments. The

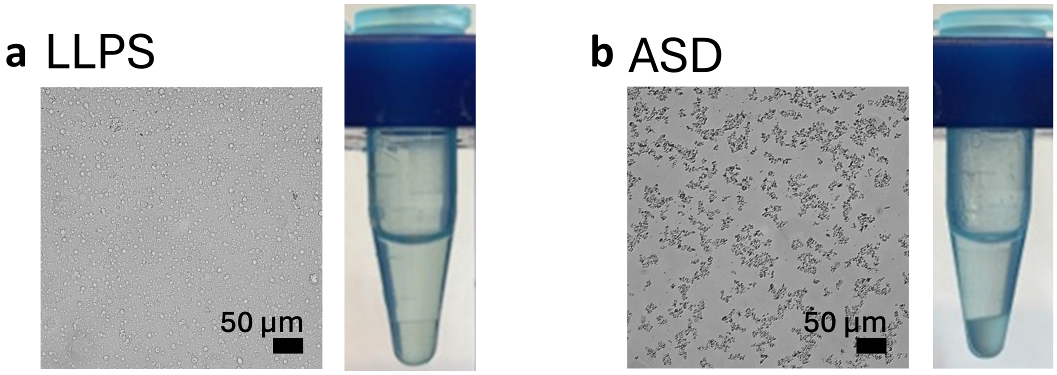


Figure S2: Brightfield microscopy images and digital camera images after centrifugation of IgG-PEG mixtures, characterized as (a) liquid-liquid phase separation (LLPS) and (b) amorphous solid dispersion (ASD).

dissolution rate of water droplets in an organic phase can generally be successfully modeled using the Epstein-Plesset (E-P) model, including with solute- and protein-containing droplets [3, 4]. The value of parameters for the E-P model in the experimental system is tabulated below in Table S1.

**Table S1.** Relevant parameters for Epstein-Plesset model.

| | |
|---|---|
| $\rho$, density of water | 0.997 g/cm$^3$ |
| f, initial saturation ratio | 0 |
| $c_s$, saturation of water in pentanol | 0.073 g/cm$^3$ [5] |
| $D$, literature diffusion coefficient | $0.52 * 10^{-5}$ cm$^2$/s [6] |

To validate the experimental setup for this work, droplets of pure water ($R_0 = 150 - 200$ $\mu$m) were generated using the setup described in the main text, and the rate of dissolution was fitted to the E-P model. The diffusion coefficient, $D$, was extrapolated from fitting the kinetic data. A correction factor of 0.72 was applied based on previous studies to account for the droplet's contact with the bottom surface of the Petri dish [7]. Using our experimental setup, we found values of the diffusion coefficient for water in pentanol ($0.50 \pm 0.04 * 10^{-5}$ cm$^2$/s) comparable to those in the literature, thus validating the current setup [6]. The data and fitted models are shown below for the validation experiments of water droplets in 1-pentanol (Figure S3). As shown, the data displayed good fit to the model, with $R^2$ values ranging from 0.933 – 0.978. In addition, when scaled by $t_f$:

$$t_f = \frac{\rho R_0^2}{2DC_s(1-f)} \tag{2}$$

which is the theoretical time for a water droplet of initial size $R_0$ to completely dissolve, the data collapse on one master curve independent of initial droplet size. Therefore, we were able to apply our fitted diffusion coefficient in the E-P model to compare to the single-droplet experiments in the main text for antibody-laden droplets.

## Single droplet experiments without surfactant

Without surfactant (Tween 80) in the pentanol phase, the antibody droplets tended to form non-spherical and irregular morphologies. A set of time-lapsed images of an antibody solution droplet in pentanol without surfactant is shown in Figure S4a. Although dehydration of the droplet and increasing PEG and IgG concentration induces precipitation of the antibody, with or without surfactant, the particle that is formed without surfactant present is misshapen with rough surface features that could affect the flowability of the particles. Therefore, surfactant was used in the pentanol phase to consistently generate smooth, spherical microparticles by controlling interfacial tension.

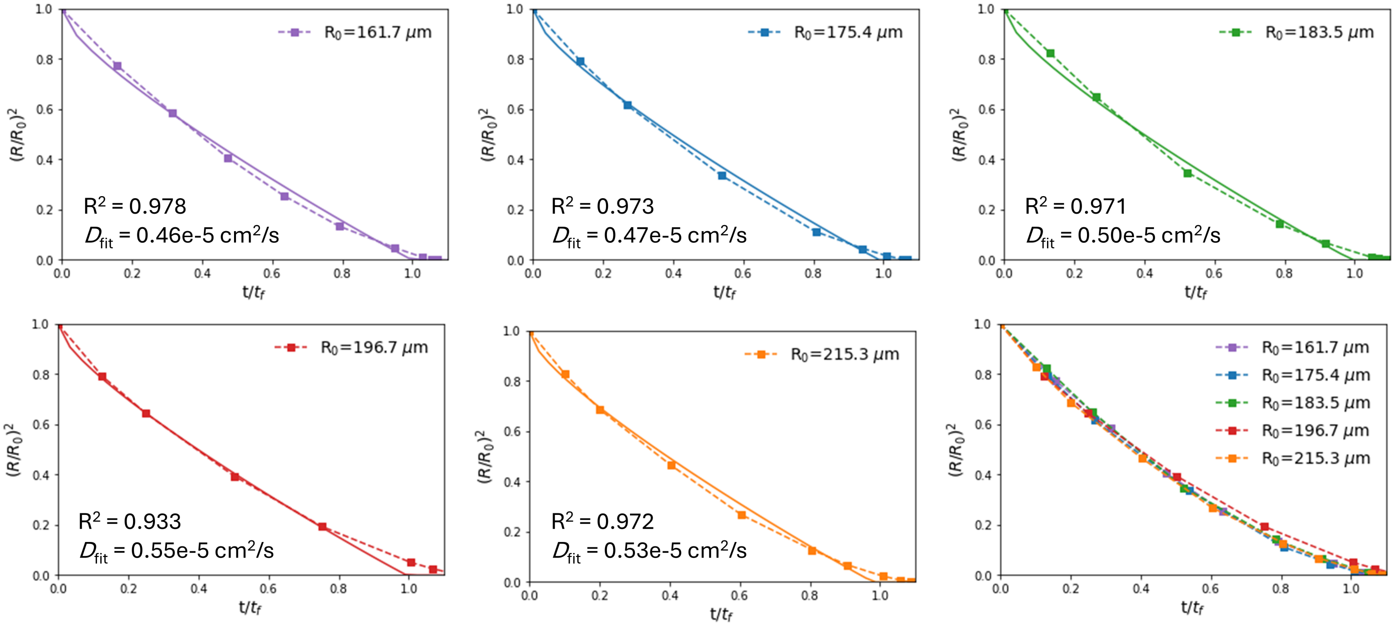


Figure S3: Scaled radius versus scaled time for the dehydration of pure water droplets in pentanol, for droplets with varying initial radii (n=5). The data were fitted to the Epstein-Plesset model to extrapolate the fitted diffusion coefficient for each condition ($D_{fit}$) [8]. R-squared values are shown for each experiment. The average of these $D_{fit}$ were taken as $D$ for further experiments. All control experiments were plotted onto a master curve.

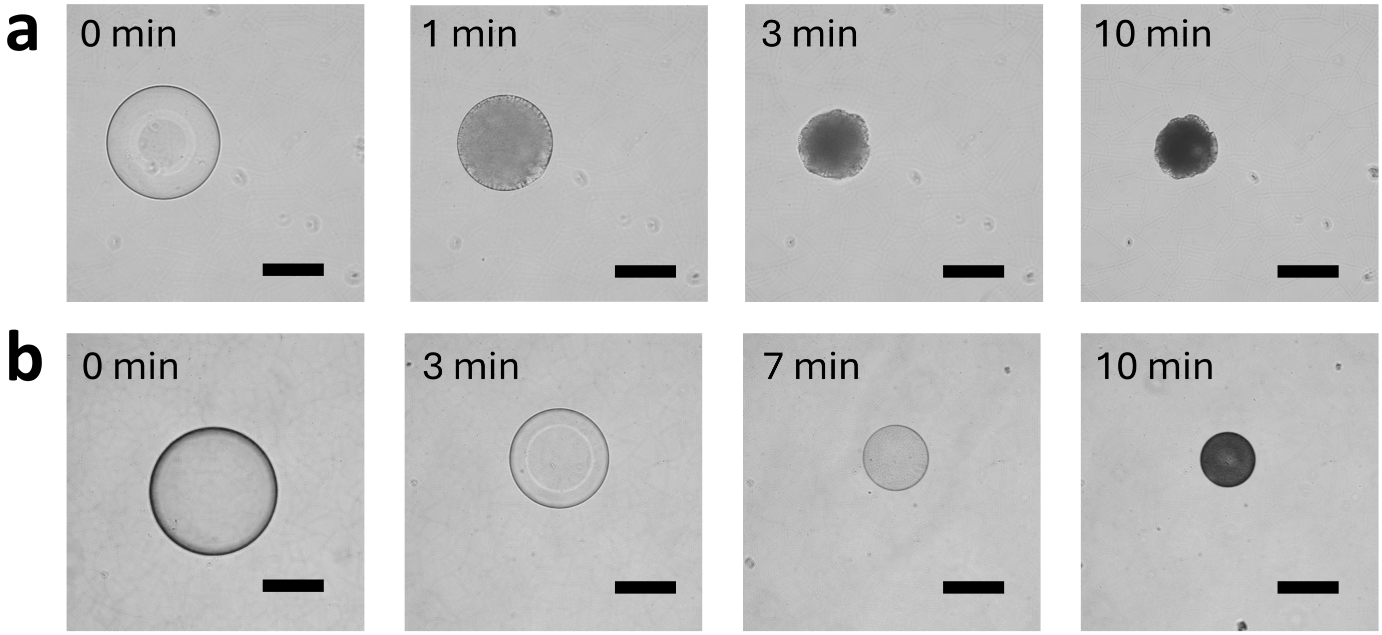


Figure S4: a) Time-lapse images of dehydration process for a droplet (60 mg/mL IgG, PEG 2% w/v), no surfactant (0% w/v Tween 80) in pentanol. b) Time-lapse images of dehydration process for a droplet without PEG (60 mg/mL IgG, 0% w/v PEG) in pentanol (0.4% w/v Tween 80). Scale bar = 200 $\mu$m.

## Single droplet experiments without PEG

As described in the main text, single droplet experiments using the validated experimental setup were performed for antibody solution droplets ($C_{IgG,o}$=60 mg/mL) without PEG (Figure S4b). Because of the absence of PEG or other polymer, the sole driving force for the antibody liquid-solid transition was the extraction of water from the droplet. During the dehydration process, the antibody is observed to remain in solution as a clear droplet until t=∼7 min, which is approximately the time it takes for the droplet to be concentrated above the solubility limit for IgG. At that time, the droplet has experienced a 300-400% decrease in its volume due to dehydration, causing IgG to reach supersaturation and precipitate out of solution into its solid form. By contrast, with PEG (2% w/v) in the initial solution, IgG begins to liquid-liquid phase separate with only a ∼30% decrease in droplet volume and fully precipitates for a ∼100% decrease in volume. Clearly, PEG when present in the initial droplet is the major driving force for protein precipitation, and it is also key for stabilizing the protein as an amorphous solid.

## Stability of antibody microparticles without alginate

As discussed in the main text, solid antibody remains encapsulated in a hydrogel microparticle in an aqueous phase due to the presence of PEG in the solution, which prevents redissolution of the antibody. This effect is consistent even without the alginate hydrogel. Particles were produced via dehydration of antibody-laden droplets in pentanol, without alginate in the initial solution, and then resuspended in a 15% w/v PEG solution. From Figure S5, we observe that the microparticles remain opaque in the aqueous PEG solution, showing that PEG is able to stabilize the solid antibody as expected, and this mechanism is not dependent on an hydrogel network.

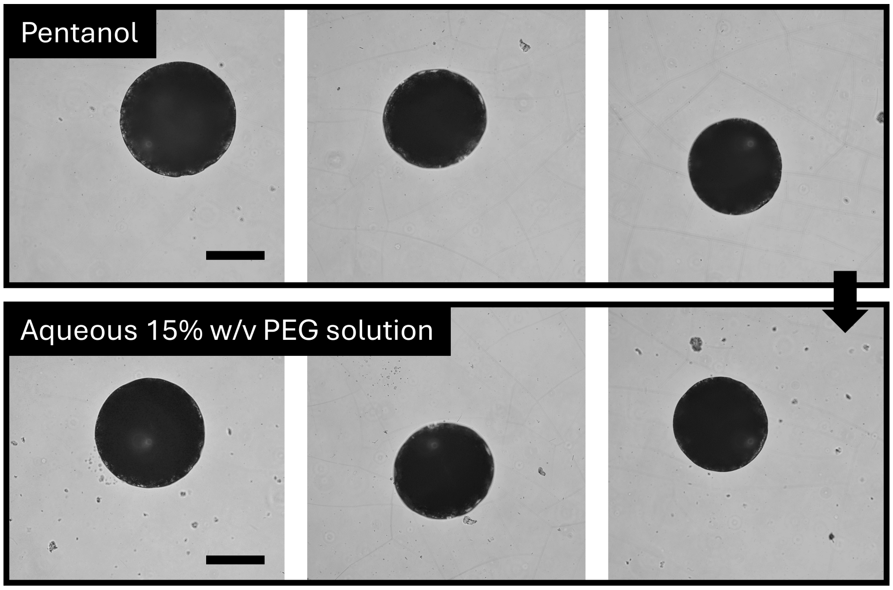


Figure S5: Images of individual ASD microparticles, without alginate, after dehydration in pentanol (top) and resuspension in 15% w/v PEG solution (bottom). Subsequent dissolution of the microparticles in simulated body fluid (SBF) was not shown due to the rapid dispersion and dissolution of the ASD in SBF. Scale bar = 100 $\mu$m.

## Estimated final particle concentration in single droplet experiments

For the single droplet experiments, the final IgG concentration ($C_{IgG,f}$) in the particle following the dehydration process could be estimated based on the observed change in droplet size and the initial IgG

concentration in the droplet. The ratio of the initial and final droplet sizes can be taken as the concentration factor:

$$C_{IgG,f} = (R_o/R_f)^3 * C_{IgG,o} \tag{3}$$

The calculated $C_{IgG,f}$ are shown in Figure S6 for various calcium concentrations in pentanol, tested in the single droplet experiments with 60 mg/mL IgG, 2% w/v PEG, and 0.2% w/v alginate initially in the droplets. Variations in $C_{IgG,f}$ between different conditions are discussed in the main text.

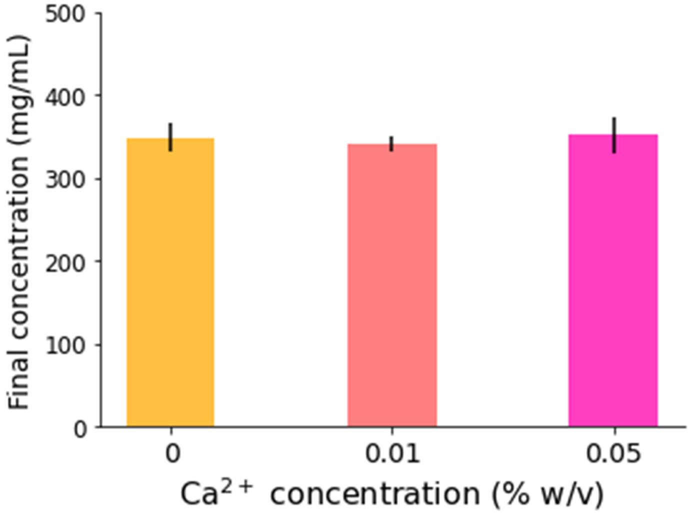


Figure S6: Final IgG concentrations (mg/mL), estimated from the initial droplet IgG concentration (60 mg/mL) and the calculated concentration factor of the droplets (n=3 for each condition), shown for varying pentanol $Ca^{2+}$ concentration (% w/v).

## FTIR spectra

Representative second derivative FTIR spectra for the amide I band region are shown below for the native reference, dehydrated particles, and the reconstituted IgG (Figure S7). The second derivative spectra were used for quantification of the antibody secondary structure, which is a standard technique for FTIR spectroscopic analysis to enhance the resolution of FTIR measurements [9].

## Enzyme-linked immunosorbent assay (ELISA)

ELISA bioassays were performed to determine the IgG antibody bioactivity using sandwich binding assays. Human IgG total kit (Invitrogen) was used for identifying IgG binding. The assay was carried out in accordance with the manufacturer's procedure. Biological activity is expressed as % relative activity of the standard sample. The samples tested were a native unprocessed control, IgG released from the particles IgG from dehydrated particles, and reconstituted IgG after formulation (orange) and after 4 months of storage (green).immediately after processing, and IgG released from the particles after 4 months of storage at $4^oC$, with each measured in triplicate (n=3). Biological activity was expressed as % relative potency of the native control sample, shown below in Figure S8.
From the bioactivity results, the potency of the IgG was not affected by the formulation process. After releasing from the hydrogel microparticles, IgG showed 100% relative potency both immediately following formulation and after 4 months of storage in the microparticle form. These results show that the antibody is functionally stable during the formulation process as well as in the long-term, which is crucial for considering clinical applications and delivery. The bioactivity results for this study were comparable to those reported in previous works with centrifugally-synthesized ASD-laden hydrogel particles, in

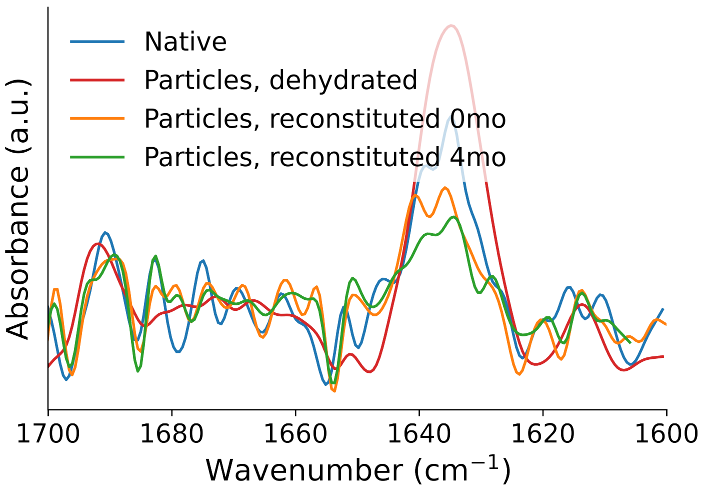


Figure S7: Representative second-derivative FTIR-ATR spectra for native IgG reference (blue), IgG from dehydrated particles, and reconstituted IgG after formulation (orange) and after 4 months of storage (green).

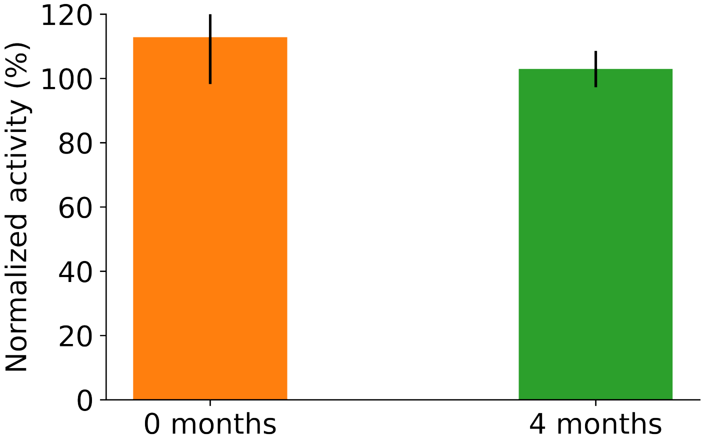


Figure S8: Normalized ELISA activity results for IgG from antibody-laden hydrogel particles, released immediately after formulation (0 months) and after 4 months of storage at 4$^{o}$C (n=3).

which IgG was stable in the particle over 100 days [10]. The complete activity of the formulated antibodies from the proposed platform suggests that the process of dehydration, precipitation, and encapsulation is compatible with the antibodies, supporting the structural analyses in the main text.

## Size exclusion chromatography (SEC)

Analytical SEC was used to determine the quantity of IgG monomer and aggregates from eluted from ASD-laden alginate microparticles. For this purpose, an Agilent 1200 HPLC instrument was used, with a TSKgel G3000SWXL (Tosoh Bioscience) analytical SEC column. SEC experiments were carried out at a flow rate of 0.4 mL/min in phosphate buffered saline (PBS) at pH 7.4. For the native control experiment, lyophilized IgG powder as received was dissolved into PBS and analyzed. The SEC results from integrating the signal are tabulated below in Table S2. Experiments were performed in triplicate (n=3) for each condition, with standard deviations reported.

**Table S2.** Stability of IgG evaluated using size exclusion chromatography.

| | Monomer % | Aggregates % |
|---|---|---|
| Native | 92.66 ± 0.39 | 7.34 ± 0.39 |
| Microparticles | 91.22 ± 0.09 | 8.78 ± 0.09 |

As seen in Table S2, the quality of the IgG released from the alginate microparticles is not significantly different from the native control (>90% monomer), indicating that IgG remains stable and does not irreversibly aggregate when formulated into the hydrogel via the solvent-based dehydration process. Characteristic UV traces for each condition are available in Figure S9.

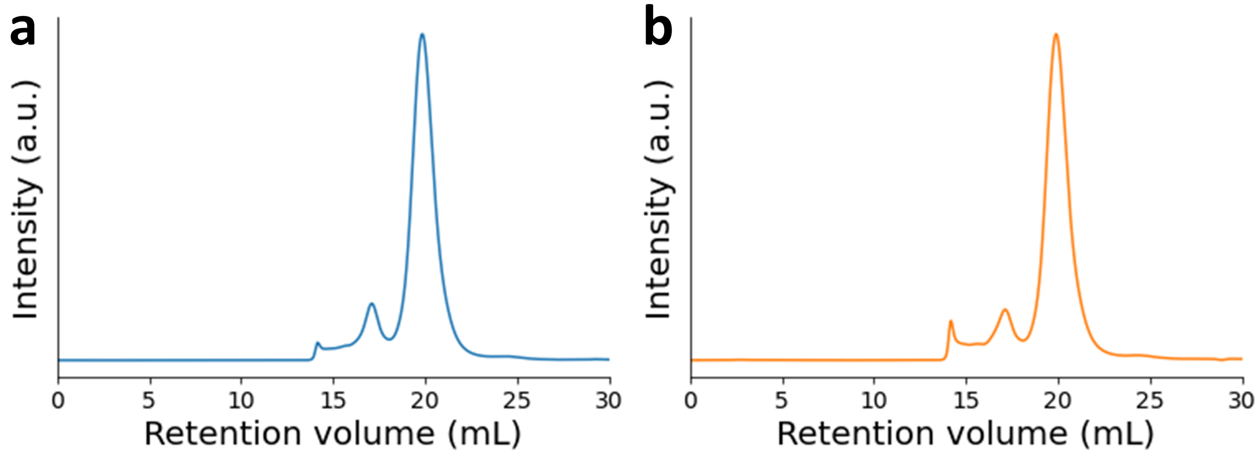


Figure S9: UV traces from size exclusion chromatography of released IgG from (a) native control sample and (b) alginate microparticles.

# References


[1] D. Atha, K. Ingham, *Journal of Biological Chemistry* **1981**, *256*, 23 12108.

[2] M. J. Scannell, M. W. Hyatt, I. L. Budyak, M. A. Woldeyes, Y. Wang, *Pharmaceutical Research* **2021**, *38*, 11 1947.

[3] D. L. Bitterfield, A. Utoft, D. Needham, *Langmuir* **2016**, *32*, 48 12749.

[4] V. N. Pham, D. Radajewski, I. Rodríguez-Ruiz, S. Teychene, *Biosensors* **2021**, *11*, 11 460.

[5] A. F. M. Barton, *Solubility Data Series*, volume 15 (Alcohols with Water), International Union of Pure and Applied Chemistry, **1984**.

[6] J. T. Su, P. B. Duncan, A. Momaya, A. Jutila, D. Needham, *The Journal of Chemical Physics* **2010**, *132*, 4 044506.

[7] S. Kentish, J. Lee, M. Davidson, M. Ashokkumar, *Chemical Engineering Science* **2006**, *61*, 23 7697.

[8] P. S. Epstein, M. S. Plesset, *The Journal of Chemical Physics* **1950**, *18*, 11 1505.

[9] J. Kong, S. Yu, *Acta Biochimica et Biophysica Sinica* **2007**, *39*, 8 549.

[10] A. Erfani, P. Reichert, C. N. Narasimhan, P. S. Doyle, *iScience* **2023**, *26*, 8 107452.
